# A Comprehensive Evaluation of Flavor Instability of Beer (Part 2): The Influence of *De Novo* Formation of Aging Aldehydes

**DOI:** 10.3390/foods10112668

**Published:** 2021-11-03

**Authors:** Arndt Nobis, Melanie Kwasnicki, Florian Lehnhardt, Michael Hellwig, Thomas Henle, Thomas Becker, Martina Gastl

**Affiliations:** 1 TUM School of Life Sciences, Technische Universität München, 85354 Freising, Germany; arndt.nobis@tum.de (A.N.); florian.lehnhardt@tum.de (F.L.); tb@tum.de (T.B.); 2 Chair of Food Chemistry, Technische Universität Dresden, 01069 Dresden, Germany; melanie_marika-elke.kwasnicki@tu-dresden.de (M.K.); thomas.henle@tu-dresden.de (T.H.); 3 Institute of Food Chemistry, Technical University of Braunschweig, 38106 Braunschweig, Germany; m.hellwig@tu-braunschweig.de

**Keywords:** beer aging, Maillard reaction, dicarbonyls, aldehydes, proteolytic malt modification, Strecker degradation

## Abstract

Flavor instability of beer is affected by the rise of aroma-active aldehydes during aging. Aldehydes can be either released from bound-state forms or formed *de novo*. This second part of our study focused on the *de novo* formation of aldehydes during the Maillard reaction, Strecker degradation, and oxidation reactions. Key precursor compounds for *de novo* pathways are free amino acids. This study varied the potential for reactions by varying free amino acid content in fresh beer using different proteolytic malt modification levels (569–731 mg/100 g d. m. of soluble nitrogen) of the used malt in brewing trials. Overall, six pale lager beers were produced from three malts (different malt modification levels), each was made from two different barley varieties and was naturally and forcibly aged. It was found that higher malt modification levels in fresh beer and during beer aging increased amino acid and dicarbonyl concentrations as aging precursors and Strecker aldehyde contents as aging indicators. Dicarbonyls were degraded during aging. Advanced glycation end products as possible degradation products showed no consistent formation during aging. Therefore, Strecker reactions were favored during beer aging. No alternative oxidative formation of Strecker aldehydes from their corresponding alcohols could be confirmed. Along with the preceding part one of our investigation, the results of this study showed that *de novo* formation and release occur simultaneously. After 4 months of natural aging, aldehyde rise is mainly accounted for by *de novo* formation.

## 1. Introduction

After bottling, beer flavor is affected by various chemical reactions, leading to flavor instability. One main reason for a change in flavor in beers is the rise of aging indicators such as sensory-active aldehydes. In our preceding study (part one), the sensory and chemical effects of beer aging in lager beer were introduced [1]. The concentration of aging-relevant aldehydes increases during storage, and these aldehydes can be either formed by their release from bound-state aldehydes forms or by *de novo* formation pathways [2]. The preceding study focused on the release of bound-state aldehydes (part one). Important reactions for *de novo* formation of aging aldehydes are the Strecker degradation, the Maillard reaction, and oxidation reactions such as lipid oxidation or oxidation of the corresponding alcohols [3,4].

**Figure 1:**
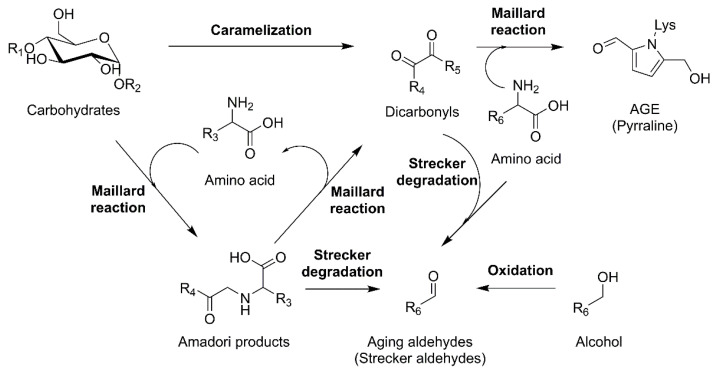
Overview of observed *de novo* formation pathways of aging aldehydes.

Despite moderate storage temperatures of approximately 20 °C, *de novo* formation of aging aldehydes can occur with low reaction rates, starting in fresh beer. Here, the concentrations of precursor substances are a critical factor for reaction kinetics [4]. Figure 1 summarizes the reaction pathways for the *de novo* formation of aldehydes observed in this study.

Regarding Strecker degradation and the Maillard reaction, amino acids, Amadori products, and dicarbonyls are important precursor substances [5,6,7]. During Strecker degradation, dicarbonyls and amino acids react to Strecker aldehydes. Here, they form an unstable hemiaminal adduct by water elimination, which further undergoes irreversible decarboxylation. After water addition, the adduct is decomposed into Strecker aldehydes and an α-ketoamine compound [4]. Important aging-relevant Strecker aldehydes are 2-methylpropanal (2MP) from valine, 2-methylbutanal (2MB) from leucine, 3-methylbutanal (3MB) from isoleucine, phenylacetaldehyde (PA) from phenylalanine, and methional (meth) from methionine. Alternatively, Strecker aldehydes could be directly formed from Amadori products [8] or by oxidation of their corresponding alcohols, e.g., 2MP from the oxidation of 2-methylbutanol [9]. Regarding the direct oxidation of higher alcohols, the pathway could be deemed insignificant due to minimal O_2_ levels after bottling (<0.1 mg/L) [10]. Further, a study on wine recently found that oxidation of higher alcohols to their respective aldehydes is significantly less relevant than the degradation of amino acids during the Strecker reaction [11].

A competing reaction pathway for dicarbonyls and amino acids is the final stage of the Maillard reaction [12]. Here, the amino groups of arginine or lysine can react with dicarbonyls forming “advanced glycation end products” (AGEs). For example, pyrraline and N^ε^-carboxyethyllysine (CEL) can be formed via the reaction of the ε-amino group of lysine with the dicarbonyl 3-deoxyglucosone (3-DG) [13] or with methylglyoxal [14], respectively. Nobis et al. demonstrated an 8% conversion from artificially spiked 3-DG to AGEs during aging [15]. Therefore, AGE formation can influence dicarbonyl reactivity during aging.

Thus far, several studies have shown a negative sensory effect of artificially increased precursors. Lund et al. pointed out that an elevated content of amino acids increased protease activity during the mashing procedure had a negative effect on beer sensory quality during aging [16]. Vesely et al. reported negative sensory effects by spiking phenylalanine and methionine to fresh beer [17]. Nobis et al. showed enhanced Strecker aldehyde formation after prolonged storage by adding 3-DG to fresh beer [15]. However, there is a lack of knowledge about the effect of technologically varied initial precursor content on *de novo* aging aldehyde formation during beer aging (forced and natural). As introduced in our preceding study (part one), accelerated proteolytic malt modification, in particular, is likely to be a good adjusting tool because it increases the levels of amino acid and dicarbonyls in malt [18] and wort [19].

In this second part of our study, we hypothesized that an increased proteolytic malt modification level elevates flavor instability by promoting *de novo* formation of aldehydes due to an enhanced initial concentration of amino acids and dicarbonyls in fresh beer. Therefore, the aim of part two was to investigate *de novo* formation pathways of aging aldehydes due to different proteolytic malt modification levels using Strecker degradation and the Maillard reaction. Furthermore, the application of precursor concentration for the early stage assessment of aging stability was evaluated.

## 2. Materials and Methods

### 2.1. Chemicals

All amino acids (L-form), (13C, 15N) labeled amino acids, D-glucose, potassium dihydrogen phosphate, methanol (LC-MS grade), o-phenylenediamine, furfural, pentanal, hexanal, heptanal, (E)-2-nonenal, hydrochloric acid, 4-fluorobenzaldehyde, ethanol (absolute), disodium hydrogen phosphate dihydrate, butanol, methyl caproate, 2-methypropanol, 3-methylbutanol, 2-methylbutanol, acetic acid, 2-methylbutyraldehyde (2MB), 3-methylbutyraldehyde (3MB), isobutyraldehyde (2MP), phenylacetaldehyde (PA), O-(2,3,4,5,6-Pentafluorobenzyl) hydroxylamine (PFBHA), nonafluoropentanoic acid (NFPA), pepsin (3839 U/mg protein), pronase E (4000 PU/mg), leucine amino-peptidase (18 U/mg protein), prolidase (553 U/mg protein), o-phenylenediamine, quinoxaline, 2-methylquinoxaline, 2,3-dimethyl-quinoxaline, and water (LC-MS grade) were obtained from Merck (Darmstadt, Germany). Acetonitrile used for liquid chromatography–mass spectrometry (LC-MS) analysis was purchased from VWR (Darmstadt, Germany). Methanol was acquired from Fisher Scientific (Loughborough, UK). Before use, the water for analytics was purified using a micropore water purification system (Thermo Fisher Scientific Inc., Waltham, MA, USA).

The following substances were synthesized according to literature: N-ε-fructosyllysine [20], N-ε-maltulosyllysine [20], pyrraline [13,21], and quinoxaline derivatives of 3-DG [22], 3-DGal [22], and 3-DM [23].

### 2.2. Malt, Wort, and Beer Production

The production of malt, wort, and beer samples has been described in our preceding study [1]. As presented in part one, the sample set comprised six beers from two barley varieties (B1 and B2) each malted at three different malt modification levels (P1 < P2 < P3) [1]. Table 1 summarizes the sample set and the malt modification level of the used malt assessed by the soluble nitrogen content [1].

For reaching the target values of soluble N, both barley varieties were malted at different steeping degrees (moisture content during germination).

### 2.3. Aging and Sample Treatment

Forced (FO) and natural beer aging from fresh beer (FR) has been described in part one of our study [1]. Sampling during natural beer aging was performed after one month (M1), two months (M2), three months (M3), four months (M4), five months (M5), six months (M6), and nine months (M9). The sampling at each sampling point was performed by filtration through a folded paper filter. After filtration, the samples were quickly homogenized, collected in 50 mL plastic tubes, and frozen. Prior to high-performance LC (HPLC) analytics, beer samples were used directly after filtration through a membrane filter (0.45 µm) from VWR (Radnor, PA, USA).

### 2.4. Quantitation of Free Aldehydes via HS-SPME-GC-MS

The procedure was performed according to Lehnhardt et al., with minor modifications [24]. The cooled wort sample (5 mL) and 50 µL internal standard (2 mg/L 4-fluorobenzaldehyde in ethanol) were transferred to a 20 mL headspace vial and stored in a cooled autosampler tray (17 °C). The extraction was performed using a Supelco^®^ 50/30 µm coating CAR-PDMS-DVB fiber from Merck (Darmstadt, Germany). First, the fiber was loaded with O-(2,3,4,5,6-pentafluorobenzyl) hydroxylamine (PFBHA) for 10 min at 40 °C. Afterward, the headspace of the sample was extracted for 30 min at 40 °C. The fiber was injected with a 1/5-split at 270 °C into a GC (GC-Ultra 1300, Thermo Scientific Inc., Waltham, MA, USA) coupled to a single quad mass spectrometer (ISQ 7000, Thermo Scientific Inc., Waltham, MA, USA). The GC was equipped with a DB-5 column (length of 60 m; inner diameter of 0.25 mm; film thickness of 0.25 µm; Thermo Scientific Inc., Waltham, MA, USA). The carrier gas was helium (flowrate 1.85 mL/min). The starting temperature was held at 60 °C for 4 min, followed by heating at 5 K/min up to a final temperature of 250 °C, which was maintained for 3 min. A full scan mode (*m/z* 35–350) with a dwell time of 0.02 s was used for the analysis. Each sample was analyzed in triplicate. Peak detection was performed in Xcalibur 3.1.66.10 (Thermo Scientific Inc., Waltham, MA, USA).

### 2.5. Quantitation of 1,2-Dicarbonyl Compounds

For this study, 1,2-dicarbonyl compounds were analyzed as quinoxaline derivatives using a high-pressure gradient system series 1200 (Agilent Technologies, Böblingen, Germany) consisting of an online degasser, autosampler, a pump, a column oven, and a diode array detector. Quantitation was performed according to Degen et al. [25].

### 2.6. Quantitation of Amino Acids

A total of 19 amino acids were determined according to the method reported by Sonntag et al. [26] by high-performance LC tandem mass spectrometry (HPLC MS/MS) in a multiple reaction monitoring (MRM) mode, as previously published by Nobis et al. [18].

### 2.7. Quantitation of Glycated Amino Acids

By using a high-pressure gradient system series 1200 (Agilent Technologies, Böblingen, Germany), including a binary pump, an online degasser, a column oven, an autosampler, and a diode array detector, as well as the triple-quadrupole mass spectrometer 6410, glycated amino acids were analyzed according to a previously published protocol [23].

### 2.8. Determination of Higher Alcohols and Esters

Higher alcohols and esters were quantified according to MEBAK WBBM 2.21.1 [27] as volatile fermentation byproducts with a gas chromatography flame ionization technique (GC-FID). The system comprised an HP 7694 Headspace Sampler and an HP 6890 Series GC from Agilent (Waldbronn, Germany). An HP-Ultra 2 capillary column (50 m × 0.32 mm × 0.52 µm) was used for chromatographic separation from Agilent. Samples were used directly without any treatment. Then, 5 mL beer and 0.1 mL of the internal standard (100 mg/L methyl caproate and 2 g/L butanol in 10 *v*/*v*% ethanol in water) were transferred in a 22 mL headspace vial and directly analyzed.

### 2.9. Statistical Analysis

Statistical analysis was performed using the software JMP^®^ Pro 14 (SAS Institute GmbH, Heidelberg, Germany). Results are presented as the average ± standard deviation. ANOVA (Tukey test) at a significance level of 0.05 was used for average comparison.

## 3. Results and Discussion

Flavor instability of beer is mainly caused by increasing aldehyde concentrations in freshly bottled beer. Since the preceding study observed the release of aging-relevant aldehydes, this study focused on *de novo* formation pathways. In the following section, the oxidation of higher alcohols, the Maillard reaction, and the Strecker degradation are discussed because of their contribution to Strecker aldehyde formation during beer aging.

### 3.1. Oxidation of Higher Alcohols

The first pathway studied is the oxidation of higher alcohols. As already introduced, these compounds can be oxidized in the presence of reactive oxygen species, forming their corresponding aldehydes [2]. The degradation is an alternative pathway for Strecker aldehyde formation. Figure S1 shows the concentrations of 2-methylpropanol (2MP precursor), 2-methylbutanol (2MB precursor), and 3-methylbutanol (3MB precursor) during natural and forced beer aging at two barley varieties (B1 and B2), each with three different malt modification levels (P1 < P2 < P3).

**Figure 2:**
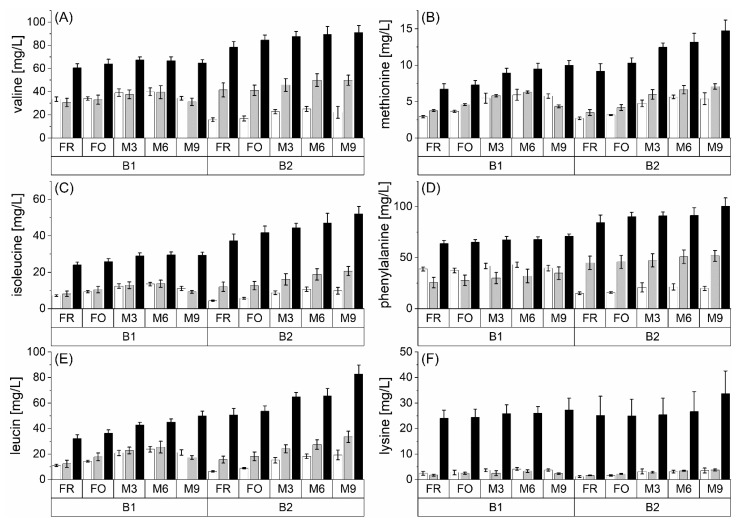
Concentrations of (**A**) valine, (**B**) methionine, (**C**) isoleucine, (**D**) phenylalanine, (**E**) leucine, and (**F**) lysine at fresh (FR), forcibly aged (FO), and naturally aged (M3, M6, and M9) beer at different malt modification levels (P1: white bar; P2: grey bar; P3: black bar) at two barley varieties (B1 and B2); *n* = 3.

There was no consistent trend in the behavior of natural beer aging at all variations. Therefore, the most constant trend did not indicate an oxidation reaction. The oxygen level was below 0.1 mg/L in fresh beer samples at all variations [1]. Thus, the oxidation reaction was inhibited and no contribution to the aging aldehyde formation could be observed. However, only the P1 levels of 2-methylbutanol and 3-methylbutanol at B2 showed a significant reduction during aging. The degradation could indicate the oxidative formation of 3MB or alternative degradation pathways of 3-methylbutanol favored such as esterification, acetal formation, or elimination reaction. Further, the aging of B2P1 is more influenced by oxygen because it showed the lowest precursor levels (Figure 2), as well as the lowest SO_2_ levels [1], but oxygen concentrations were low, compared with the other variations in this study. Regarding the effect of malt modification, there was also no consistent trend observable. Surprisingly, the medium malt modification level (P2) showed the highest concentrations for 2-methylpropanol and 2-methylbutanol. The higher alcohol concentration measured in fresh beer is mainly formed during fermentation from their corresponding amino acids via the Ehrlich pathway [28]. Although the highest amino acid concentrations of valine, leucine, and isoleucine were found in the highest malt modification level (P3) after boiling [19], a differentiation of the corresponding higher alcohols due to the order in boiled wort (P1 < P1 < P3) was not detected. That confirms that the Ehrlich pathway is affected by various parameters such as yeast strain, pH value, aeration, or additional glucose content, which was not monitored in this study, in boiled wort [28,29]. Exemplarily, 3-methylbutanol showed no trend within the different malt modification levels.

Generally, there was no consistent indication for oxidative Strecker aldehyde formation during beer aging for the observed analytes in this study, which confirms the literature study on wine [11], as already elucidated in the Introduction Section.

### 3.2. Reactivity of Precursor Compounds in the Maillard Reaction

The possible precursor compounds for flavor-active Strecker aldehydes are primarily the corresponding amino acids and dicarbonyl compounds. The latter can be formed because of direct caramelization, or indirectly via the Amadori product degradation as part of the Maillard reaction from carbohydrates [5]. Figure 2 shows the free amino acids depending on the malt modification level (P1 < P2 < P3) for two barley varieties (B1 and B2) during natural and forced aging. Valine, methionine, leucine, isoleucine, and phenylalanine were selected because their corresponding Strecker aldehydes are sensory-active beer aging indicators. Furthermore, lysine is shown to be an important reactive and often modified amino acid within the Maillard reaction.

The highest contents of the selected amino acids were determined at the highest malt modification level. The observed effect confirmed the ratio of the amino acids in the final wort [19]. Although amino acids were metabolized during yeast fermentation [9], their ratio between different malt modification levels remained constant. It is evident that this ratio differed between the observed amino acids. Thus, the difference in free lysine concentration between the lowest and highest malt modification level in fresh beer was at factor 20, whereas methionine and valine concentrations showed factor 2–5. This may be due to biological differences in protein composition and enzyme activity of barley varieties [30]. Generally, an increased malt modification level provides higher reaction potential for *de novo* Strecker aldehyde formation due to increased amino acid content.

The forced aging of the beers did not lead to significant changes in amino acid concentrations. This is partly due to the low reactivity of amino acids and, further, the relatively short reaction time of 4 days, compared with natural aging. However, natural aging increased Strecker-active amino acids. This could be explained, on the one hand, by the release from imines or Amadori products and, on the other hand, the degradation of peptides and proteins. Thus, it seems likely that the degradation of Strecker aldehydes occurred simultaneously with the release of amino acids. Therefore, an increasing amino acid concentration during aging could additionally increase the *de novo* formation potential for Strecker aldehydes.

**Figure 3:**
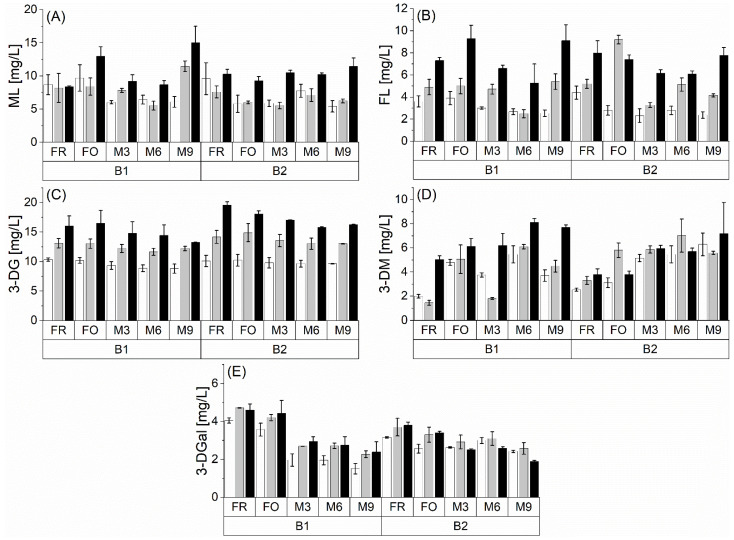
Concentrations of Amadori products ((**A**) ML and (**B**) FL) and dicarbonyls ((**C**) 3-DG, (**D**) 3-DM, and (**E**) 3-DGal) at fresh (FR), forcibly aged (FO), and naturally aged (M3, M6, and M9) beer at different malt modification levels (P1: white bar; P2: grey bar; P3: black bar) at two barley varieties (B1 and B2); *n* = 3.

Additional precursor compounds that can be formed during the Maillard reaction are Amadori products and the resulting dicarbonyl compounds. Strecker aldehydes can be formed from both substance classes [7]. Exemplarily, the Amadori products of lysine (ML and FL) and the dicarbonyl compounds 3-DG, 3-DGal, and 3-DM are discussed. Figure 3 shows the mentioned analytes in their free form, depending on the malt modification level (P1 < P2 < P3), at two barley varieties (B1 and B2) during natural and forced aging.

FL ranged from 3.0 to 11.4 mg/L. ML was determined at 5.5–15.0 mg/L. No significant differences could be seen between the barley varieties with regard to the Amadori product concentration. During forced and natural beer aging, there was also no consistent trend of the Amadori product levels. Note that this study focused on ε-terminal Amadori products of lysine (FL and ML). The α-terminal Amadori products of Strecker-active amino acids might undergo degradation and, therefore, can contribute to Strecker degradation [7]. Regarding the influence of the malt modification level, the highest level mainly showed the highest concentrations of Amadori products. The effect could be caused by the increased concentration of amino acids (Figure 2), which was already observed during wort boiling [19]. This fact indicates that the FL and ML levels could have already been increased in the final wort before fermentation.

As main dicarbonyl compounds, 3-DG (7.9–19.5 mg/L), 3-DGal (1.5–4.7 mg/L), and 3-DM (1.5–9.9 mg/L) were found in concentrations comparable to previous studies [31,32]. B2 showed slightly higher contents of dicarbonyl compounds. Regarding the 3-DG and 3-DGal content, a decreasing trend during natural beer aging could be observed, as has been previously reported [15,31]. This decrease is caused by the degradation of dicarbonyls, indicating the *de novo* formation of Strecker aldehydes. Furthermore, dicarbonyl compounds can undergo conversion to 5-hydroxymethylfurfural (HMF), as previously reported during beer aging [15]. Nevertheless, the formation of both dicarbonyls cannot be excluded during aging. Exemplarily, Rakete et al. observed the formation of 3-DG and 3-DGal by forced aging model experiments of maltotetraose [31]. Therefore, the degradation trend of both compounds tends to be underestimated. A higher malt modification level showed a consistent increase in 3-DG concentration during natural and forced aging, whereas 3-DGal content was less affected. However, higher 3-DG content could be caused by a stronger formation during wort boiling at higher malt modification levels [19,33]. The resulting higher absolute degradation of 3-DG promotes the *de novo* formation of Strecker aldehydes during natural beer aging. Furthermore, this effect can decrease flavor instability, as already shown by artificially increased 3-DG content in pale lager beers [15]. Dicarbonyl 3-DM showed a slightly increasing tendency during natural beer aging. This could be caused by a new formation from maltose. This phenomenon has already been described in the aging of pilsner beer [31]. Thus, the participation of 3-DM in Strecker degradation can be confirmed, but the degradation of α-dicarbonyl can occur simultaneously with the dominant formation during beer aging.

In summary, no clear indication for the formation of Strecker aldehydes from 3-DM and Amadori products could be observed. It should be considered that only free dicarbonyls were used for the observation and that only ε-terminal Amadori products were quantified. In contrast, the occurring degradation of 3-DG and 3-DGal indicated a possible *de novo* formation of Strecker aldehydes.

### 3.3. Formation of Free Aging Aldehydes during Beer Aging

**Figure 4:**
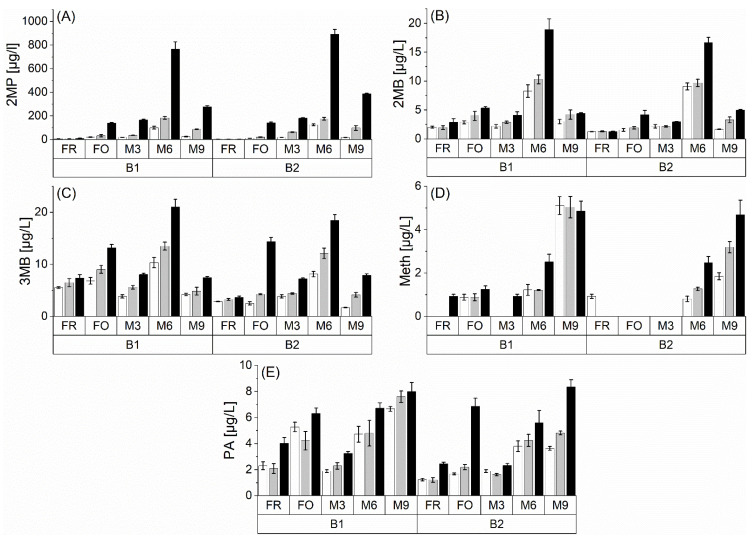
Concentrations of free Strecker aldehydes ((**A**) 2MP, (**B**) 2MB, (**C**) 3MB, (**D**) Meth, and (**E**) PA) at fresh (FR), forcibly aged (FO), and naturally aged (M3, M6, and M9) beer at different malt modification levels (P1: white bar; P2: grey bar; P3: black bar) at two barley varieties (B1 and B2); *n* = 3.

The elucidated precursor compounds react further during beer aging and form aging aldehydes, causing sensory deterioration. This study focused on the Strecker degradation reaction because this is a typical reaction of the observed precursors amino acids, dicarbonyl compounds, and Amadori products. Figure 4 shows the concentration of 2MP, 2MB, 3MB, meth, and PA during natural and forced aging in two barley varieties (B1 and B2) and three different malt modification levels (P1 < P2 < P3). Data of all sampling points are provided in the Supplementary Excel file (Table S1).

The barley variety showed no effect. The formation of the aging aldehydes was comparable for both barley varieties. During aging, aldehydes were formed. As already shown in our preceding study, the rise was caused by the release from bound-state forms [1]. However, due to the observed decreasing levels of 3-DG and 3-DGal as Strecker aldehyde precursors during aging, *de novo* formation was suggested to be occurring simultaneously. In what follows, the aging behavior of the observed Strecker aldehydes is discussed separately.

The 2MP concentration showed a strong increase during natural and forced aging. The concentration after forced aging represented levels comparable to the natural aging of 0–3 months. During natural aging, the 2MP concentration increased up to 6 months and was then reduced up to 9 months. A shift to masked forms can be excluded because, after 9 months of natural aging, no bound-state form was observed in the preceding study [1]. It can be assumed that aldehydes are degraded by alternative reactions such as aldol condensation or further oxidation of the carbonyl group. Furthermore, 2MP surpassed its threshold (86 µg/L) [34] after 3 months of natural aging at the highest malt modification level (P3) and after 5 months at low (P1) and medium (P2) levels. Sensory attributes showed significant changes after natural aging ranging from 3 to 5 months [1]. The conformity indicates that 2MP played a key role in the sensory changes during beer aging in the study. It is worth noting that 2MB and 3MB were also formed during natural and forced aging but less strongly than 2MP. The concentration of both compounds after forced aging was comparable to the concentration after 3–6 months of natural aging. As already discussed for the 2MP concentration, the degradation of both aldehydes occurred from 6 to 9 months of natural aging. This effect could also be caused by alternative degradation pathways such as aldol condensation or oxidation reactions. In this study, 2MB and 3MB did not surpass their flavor thresholds (2MB: 45 µg/L; 3MB: 56 µg/L) [34] but could still contribute to sensory flavor changes by synergistic effects. The limit of detection of meth is 0.8 µg/L. Therefore, the Strecker aldehyde was majorly quantifiable after forced aging in B1 and after 3 months of natural aging in both barley varieties. For meth, forced aging was comparable to the natural aging of 3–6 months. After 3 months of natural aging, a constant increase in meth concentration was observed. The aldehyde surpassed its threshold of 4.2 µg/L [34] after 9 months of natural aging. This result indicates that meth critically contributes to sensory deterioration in an advanced stage of beer aging (>6 months of natural aging). PA concentration increased constantly after 3 months of natural beer aging. The forced method yielded PA concentrations comparable to 6–9 months of natural beer aging, with an exception of B2P1 and B2P2 (3–6 months of natural beer aging). The aldehyde did not surpass its threshold at 105 µg/L [34] but could contribute to sensory deterioration by synergistic effects such as 2MB and 3MB.

Generally, an increasing malt modification level caused an enhanced formation of all Strecker aldehydes during natural and forced aging. This effect indicated an accelerated beer aging by both increased release and *de novo* formation of Strecker aldehydes at higher malt modification levels. Therefore, *de novo* formation is promoted by a higher content of amino acids and dicarbonyls as Strecker aldehyde precursors due to an increased modification level, as presented in the previous section.

### 3.4. Formation of AGEs

**Figure 5:**
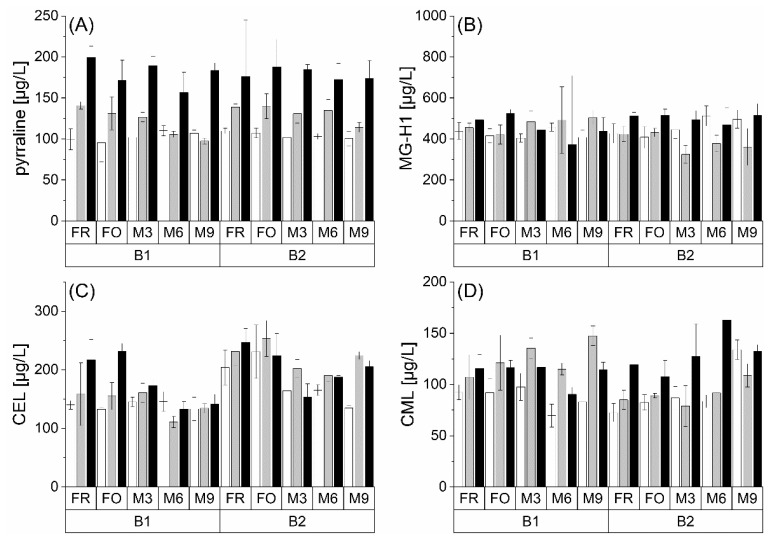
Concentrations of free AGEs ((**A**) pyrraline, (**B**) MG-H1, (**C**) CEL, and (**D**) CML) at fresh (FR), forcibly aged (FO), and naturally aged (M3, M6, and M9) beer at different malt modification levels (P1: white bar; P2: grey bar; P3: black bar) in two barley varieties (B1 and B2); *n* = 3.

Glycated amino acids are formed in the late phase of the Maillard reaction and are considered their end products [12]. Since the formation also occurs in dicarbonyl compounds, it represents a concurrence reaction to the Strecker degradation. In addition to pyrraline, AGEs MG-H1, CEL and CML were investigated. Here, only the free amino acids were determined. Figure 5 shows the contents of these AGEs depending on the malt modification level (P1 < P2 < P3) in two barley varieties (B1 and B2) during natural and forced aging.

Pyrraline, formed from 3-DG and lysine, was analyzed within 81–188 µg/L. The reaction of MGO with the guanidino group of arginine produces MG-H1, which was quantified at 325–515 µg/L in all samples. In comparison to previous studies, both concentration ranges could be considered low [15,35]. CEL and CML are derived from the reaction of MGO and GO with lysine, and thus, similar to MG-H1, are only indirectly involved in the degradation of 3-DG. CEL ranged between 111 and 253 µg/L, and CML was found between 69 and163 µg/L. Here, the contents were in the range described for pale lager beer [15].

In general, no influence of barley variety on the investigated AGEs could be observed. Higher malt modification tended to result in higher content of AGEs. Natural and forced beer aging revealed no consistent trend on the analytes. This confirmed the results of a study by Nobis et al., in which the concentration of AGEs also remained constant during beer aging. The authors suspected AGE formation during beer aging as a minor reaction [15]. Furthermore, Rakete et al. observed only a minimal increase in AGEs derived from proline (N-formylproline and N-carboxymethlyproline) during a forced aging experiment [31]. Thus, the formation of the selected AGEs from the precursor compound 3-DG could be considered as subordinate.

### 3.5. Influence of the Proteolytic Malt Modification Level on De Novo Formation

**Figure 6:**
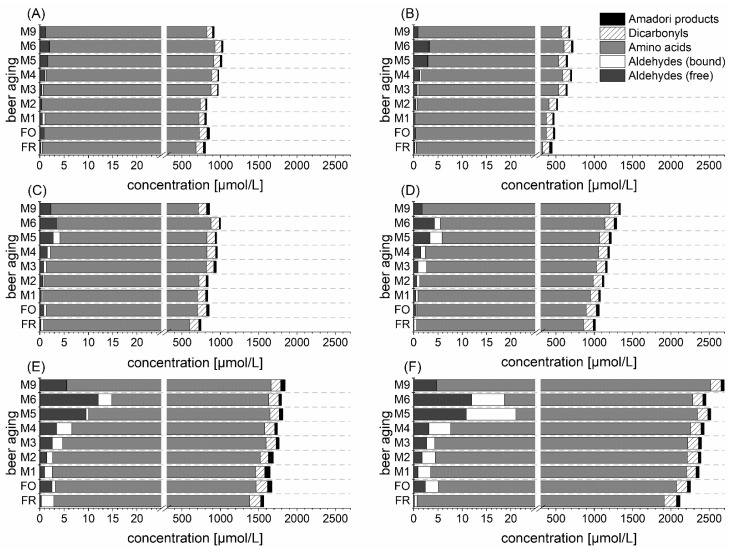
Molar distribution of free and bound aldehydes and precursor compounds (amino acids, dicarbonyls, Amadori products) during aging in beer at three different malt modification levels (P1 < P2 < P3) and two barley varieties (B1 and B2); (**A)** B1P1; (**B**) B2P1; (**C**) B1P2; (**D**) B2P2; (**E**) B1P3; (**F**) B2P3.

The previous sections focused on several individual precursor compounds and aging aldehydes. However, the contribution to *de novo* formation should be further regarded for each substance class in a holistic perspective. Hereby, the influence of malt modification level on the aging potential is focused in the following lines. Figure 6 shows the molar distribution of the sum of free aldehydes, bound aldehydes (4-VP releasable aldehydes) [1], and precursor compounds (amino acids, dicarbonyls, and Amadori products) during aging due to different malt modification levels (P1 < P2 < P3) and barley varieties (B1 and B2). For amino acids, the sum of Strecker-relevant amino acids (leucine, isoleucine, valine, methionine, and phenylalanine) was calculated. Dicarbonyls comprised the sum of 3-DG, 3-DGal, and 3-DM concentrations. Amadori products were calculated as the sum of FL and ML content. All three substance classes are precursor compounds contributing to Strecker degradation. The 4-VP releasable aldehydes from the preceding article were included in the evaluation to further elucidate comprehensively the influence of *de novo* formation and release for the formation of flavor-active volatiles. All observed compounds contribute to the actual aging potential at several aging stages [19].

In what follows, all substance classes are considered, starting with precursors. Strecker-active amino acids represented the highest percentage of the overall sum of the calculated compounds. Notably, 73–93% of the sum belonged to this substance class. Furthermore, the concentration of amino acids was 5–16 times higher than that of dicarbonyls, accounting for only 5–20% of the aging potential (sum of all compounds). The ratio of amino acids and α-dicarbonyls indicated that the dicarbonyl concentration was the critical factor for *de novo* Strecker degradation from both classes. This fact is further substantiated by the higher reactivity of dicarbonyl compounds [5]. Dicarbonyls were the only precursor substance class in which degradation was observed for almost all variations. This fact could be shown by the changes in the sum from fresh beer to 9 months naturally aged beer (B1P1: 95 to 75 µM; B1P2: 114 to 103 µM; B1P3: 143 to 120 µM; B2P1: 90 to 94 µM; B2P2: 120 to 113 µM; B2P3: 156 to 134 µM). The degradation indicated Strecker degradation and confirmed previous references that also observed dicarbonyl degradation during beer aging [15,36]. The Amadori products accounted for only a minor percentage of 1–8% of the aging potential at all aging stages. Here, it should be considered that only two Amadori products as direct precursors of dicarbonyls were observed. Amadori products formed at the α-amino group of amino acids as direct Strecker aldehyde precursor compounds [8] might be more important. For example, Wittmann and Eichner (1989) determined fructose-derived Amadori products from valine, leucine, and isoleucine in the sum of 105 µM in fresh beer [37].

Regarding free and bound-state aldehydes, it was revealed that both forms in sum accounted for only 0.7% of the aging potential. The precursor substance classes were highly elevated, compared with the free aldehydes in this study, whereby the ratio increased toward free aldehydes during aging. In fresh beer, the precursor concentration (sum of amino acids, dicarbonyls, and Amadori products) was 3050–13,790 times higher than free aldehyde concentration. After natural aging of 9 months, the ratio decreased to only 334–787 times. The shift and ratio indicate that *de novo* formation of aging aldehydes can occur directly starting from freshly bottled beer. The concentration of bound aldehydes increased with higher amino acid concentration, indicating imine formation.

The malt modification level had a strong influence on the entire aging potential. During all stages of natural and forced aging, the sum of free aldehydes, bound-state aldehydes, and precursors was increased by a higher malt modification level. The difference between B1P1 and B1P2 was low because both samples had the lowest difference between the soluble nitrogen content of the used malts [1]. However, it could be stated that a higher proteolytic malt modification level increases the *de novo* formation of free aging aldehydes by an elevated pool of precursors in fresh beer.

Regarding the result of the preceding article [1] and this study, it could be concluded that the release of the bound-state and *de novo* formation of aging aldehydes occurred simultaneously. However, both studies aimed to comprehensively elucidate whether one of both pathways was dominant at a certain aging period. Therefore, a calculation was performed to evaluate the importance of the release of aging aldehydes. The sum of the concentrations of free and bound (4-VP releasable) aging aldehydes [1] in fresh beer was compared with the concentration of free aldehydes of the following sampling points during natural aging for each barley variety and malt modification level. The aging period, where the calculated sum in fresh beer exceeded the concentration of free aldehydes in the aged beer, was defined as the release-dominated stage. Neglecting the simultaneously occurring *de novo* formation, within these months of aging, the free aldehydes were mainly formed by their observed bound-state concentrations in fresh beer. Table 2 summarizes the release-dominated aging period of each variation. Longer ranges indicate that *de novo* formation of aging aldehydes is less important.

A higher malt modification level at B2 tended to favor earlier importance of *de novo* formation pathways, whereas no influence was observed for B1 variations. Generally, it could be shown that the 4-VP releasable bound-state aldehyde was predominant toward aging aldehyde formation up to 4 months of natural aging. The result confirmed the findings of the study by Nobis et al., in which Strecker degradation by 3-DG spiking was observable after prolonged storage (>6 months) [15]. The sample set showed varied release-dominated aging periods. Therefore, *de novo* formation became important starting from 1 to 4 months of natural aging in this study.

## 4. Conclusions

The objective of the study was to investigate the *de novo* formation of free aldehydes during beer aging due to different malt modification levels. Thus, the investigation focused on the oxidation of higher alcohols, Strecker degradation, and the Maillard reaction. No formation of Strecker aldehydes from their corresponding alcohols could be confirmed. A higher malt modification level increased the concentration of amino acids, dicarbonyls, and free Strecker aldehydes. This increased aging potential explains the decreased sensory evaluation in the preceding article by a higher malt modification level [1]. The dicarbonyl compounds 3-DG and 3-DGal were degraded during aging, indicating Strecker degradation. Amadori products showed no consistent trend during aging. The effect might be caused by a balance between aldehyde formation and degradation, which could further lead to observed increased 3-DM concentrations during aging. AGEs remained constant during beer aging. Therefore, dicarbonyls reacted more strongly to Strecker aldehydes or alternative pathways such as HMF or melanoidin formation. For Strecker degradation, dicarbonyl concentrations were found to be more critical than the amino acid concentration. Finally, it was concluded that *de novo* formation and release of bound-state aldehydes occurred simultaneously. Up to four months of natural aging, the release of bound-state aldehydes was the predominant way for aging aldehyde formation. Significant sensory changes occurred at a range of 3–5 months of natural aging. Therefore, it can be assumed that the release of aldehydes basically provided the potential for sensory changes and needs to be regarded as a separate aging mechanism. In conclusion, *de novo* formation mainly reinforced and accelerated sensory beer aging after 3–5 months in this study.

## Figures and Tables

**Table 1:** Malt variation of the sample set: steeping degree, soluble N (target and real) [1].

Malt Variation	Steeping Degree (%)	Target Soluble N (mg/100 g malt d. m.)	Real Soluble N (mg/100 g malt d. m.)
B1P1	38	550 ± 25	573 ± 10
B1P2	41	625 ± 25	601 ± 1
B1P3	44	700 ± 25	660 ± 1
B2P1	39	550 ± 25	569 ± 3
B2P2	43	625 ± 25	620 ± 14
B2P3	47	700 ± 25	731 ± 1

**Table 2:** Release dominated aging period of aldehydes of pale lager beer from two barley varieties (B1 and B2) and three different malt modification levels (P1 < P2 < P3).

Variation	Release Dominant Period of Natural Aging
B1P1	0–4 months
B1P2	0–4 months
B1P3	0–4 months
B2P1	0–3 months
B2P2	0–2 months
B2P3	0–1 months

## Data Availability

The data presented in this study are available within the article.

## Supplementary Materials

The following are available online at https://www.mdpi.com/article/10.3390/foods10112668/s1, Figure S1: Concentrations of (A) 2-methylpropanol, (B) 2-methylbutanol, and (C) 3-methylbutanol in fresh (FR), forcibly aged (FO), and naturally aged (M3, M6, and M9) beer at different malt modification levels (P1: white bar; P2: grey bar; P3: black bar) at two barley varieties (B1 and B2); n = 3, Table S1: All data from amino acids, aldehydes, Amadori products, dicarbonyls, and AGEs.
