# Supplementary material for: A Comprehensive Evaluation of Flavor Instability of Beer (Part 2): The Influence of De Novo Formation of Aging Aldehydes
Source: Foods. 2021 Nov 3;10(11):2668. doi: 10.3390/foods10112668 (PMC8622366; doi:10.3390/foods10112668)
Supplement: Supplementary file 1 [file foods-10-02668-s001.zip › Supplementary Materials-Figure S1.pdf]

Supplementary Materials

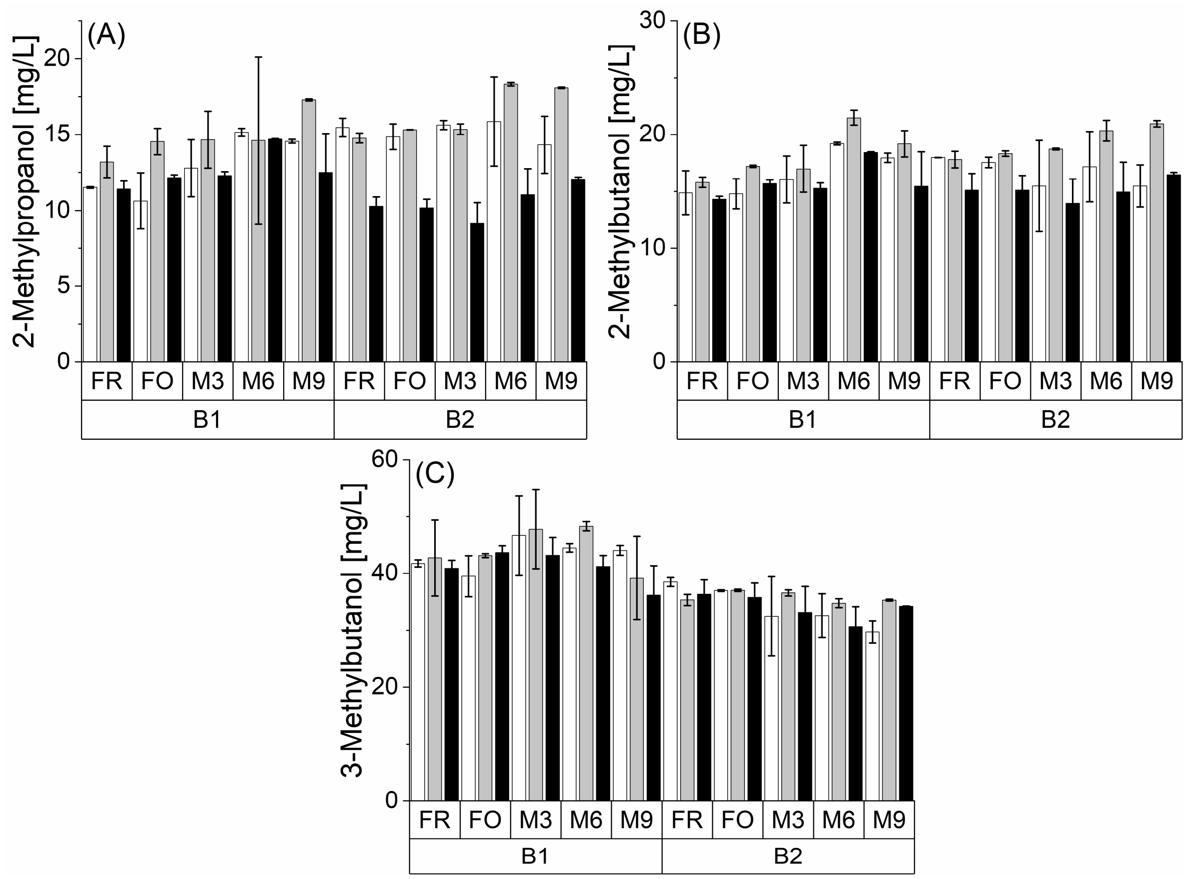

Figure S1: Concentrations of (A) 2-methylpropanol, (B) 2-methylbutanol, and (C) 3-methylbutanol in fresh (FR), forcibly aged (FO), and naturally aged (M3, M6, and M9) beer at different malt modification levels (P1: white bar; P2: grey bar; P3: black bar) at two barley varieties (B1 and B2); *n* = *3*,
